# Comparison of Movement of the Upper Dentition According to Anchorage Method: Orthodontic Mini-Implant versus Conventional Anchorage Reinforcement in Class I Malocclusion

**DOI:** 10.5402/2011/321206

**Published:** 2010-12-23

**Authors:** Ah-Young Lee, Young Ho Kim

**Affiliations:** Department of Orthodontics, The Institute of Oral Health Science, Samsung Medical Center, Sungkyunkwan University School of Medicine, No. 50, Irwon-dong, Gangnam-Gu, Seoul 135-710, Republic of Korea

## Abstract

*Objective*. To compare the amounts of anchorage loss in the upper first molar (U6) and of retraction of the upper central incisor (U1) in cases with Class I malocclusion between orthodontic mini-implants (OMIs) and conventional anchorage reinforcements (CARs). *Methods*. The subjects were 40 female adult patients with Class I malocclusion who were treated with extraction of the first premolars and sliding mechanics. The subjects were divided into Groups 1 (*N* = 20, CAR) and 2 (*N* = 20, OMI) according to anchorage method. Lateral cephalograms were taken before (T0) and after treatment (T1). Seven skeletal and dental variables and ten anchorage variables were measured. Mann-Whitney test was used for statistical analysis. *Results*. Group 2 showed significantly larger retraction of U1 (U1E-sag, 9.5 mm : 7.1 mm, *P* < .05) and less anchorage loss of U6 (U6M-sag, 0.2 mm : 2.2 mm, *P* < .05; U6A-sag, 0.3 mm versus 2.4 mm, *P* < .01) than Group 1. There was opposite vertical movement in U1 and U6 between Groups 1 and 2 (U1E-ver, 0.9 mm intrusion : 0.7 mm extrusion; U6F-ver, 1.0 mm intrusion : 0.9 mm extrusion, *P* < .05). *Conclusion*. Although OMI could not reduce the treatment duration, it could provide better maximum anchorage of U6, greater retraction of U1, intrusion of U1 and U6 than CAR.

## 1. Introduction

Generally, the common method for straightening the patient's convex profile in cases with bimaxillary dentoalveolar protrusion is orthodontic treatment with an extraction of the upper and lower first premolars and a retraction of the anterior segments under maximum anchorage [1–3]. Maximum anchorage means less than 25 per cent of space closure in the extraction space via posterior anchorage loss [4]. Therefore, an accurate prediction of the amount of anchorage loss during retraction of the anterior teeth is critical for treatment planning and selection of the appropriate mechanics.

Traditionally, conventional anchorage reinforcements (CARs), such as cervical or high pull headgear (HG) and/or transpalatal arch (TPA), have been used for this purpose. Recently, there has been a dramatic increase in the use of orthodontic mini-implants (OMIs), (also known as temporary anchorage devices (TADs)) to allow maximum anchorage, decrease the need for patient compliance, and simplify the treatment procedure [5–12]. 

Although there are several reports on the comparative evaluation between skeletal anchorage and conventional anchorage during an *en masse* retraction of the anterior teeth [13–16], the subjects in these studies were heterogeneous due to use of patients with Class II malocclusion and Class I malocclusion/bimaxillary dentoalveolar protrusion. In cases with Class II malocclusion, the correction of Class II molar relationship to Class I key requires distalization of the upper molars or mesialization of the lower molars. Hence, the effect of anchorage preservation of the posterior teeth cannot be compared in combined samples with Class II malocclusion and Class I bimaxillary dentoalveolar protrusion. 

Therefore, this study compared the anchorage loss of the upper posterior teeth and the amount of retraction of the upper anterior teeth in Class I malocclusion patients with bimaxillary dentoalveolar protrusion and minimal crowding between CAR and OMI. The null hypothesis was that there would be no significant differences in the anchorage loss of the posterior teeth and the amount of retraction of the anterior teeth using either OMI or CAR.

## 2. Materials and Methods

The initial sample in this retrospective study consisted of Korean female young adult patients (*N* = 125; mean age = 23.32 years; range = 18 ~ 35 years) who had Class I malocclusion with dentoalveolar protrusion and minimal crowding that required maximum posterior anchorage. The final samples (*N* = 40, Table 1) were selected from the initial ones according to the following criteria:

- cases with craniofacial deformities including a cleft lip and palate and other syndromes, facial asymmetry (chin point deviation >4 mm), and supernumerary or more than two missing teeth were excluded;
- women older than 17 years to eliminate the gender- and growth-related bias;
- class I molar relationship, normal overbite (>0 mm, <4 mm), labioversed upper incisor (U1 to FH plane >115°), minimal crowding in each arch (<4 mm), and a skeletal Class I malocclusion (0° < *ANB* < 4°);
- lip protrusion (lower lip to Ricketts' esthetic line >2 mm);
- treatment method.
  1. Fixed appliance and archwire: MBT brackets (.022′′ slot, 3M-Unitek, Monrovia, CA, USA) with .019 × .025 inch stainless steel wire for *en masse* retraction of the upper anterior teeth.
  2. In Group 1 (*N* = 20), the upper and lower first premolars were extracted, CAR, such as HG (12–14 hours/day, 350 gram/side) and TPA were applied, and the sliding mechanics described in the MBT technique [17–19] were performed. All the patients with HG showed good compliance by the doctor's instruction.
  3. In Group 2 (*N* = 20), the upper and lower first premolars were extracted, an OMI (Dual-Top Anchor system, Jeil Medical Co, Seoul, Korea; 1.6 mm diameter, 8 mm length, self-drilling type) was applied and the sliding mechanics were performed. The OMI was placed in the buccal attached gingiva areas between the upper second premolar and first molar adjacent to the mucogingival junction of the upper arch after leveling and alignment. Ni-Ti closed coil springs (medium, 9 mm, Jinsung, Seoul, Korea) stretched from the OMI head to the hook on the archwire between the upper lateral incisor and canine were used from two weeks after OMI installation. If the OMI was unstable or failed, a new one was installed at the buccal attached gingiva between the upper first and second molars.
- Treatment results.
  1. Finished with a Class I canine and molar relationship.
  2. Normal overbite and overjet (>2 mm and <4 mm, resp.).

**Figure 1:**
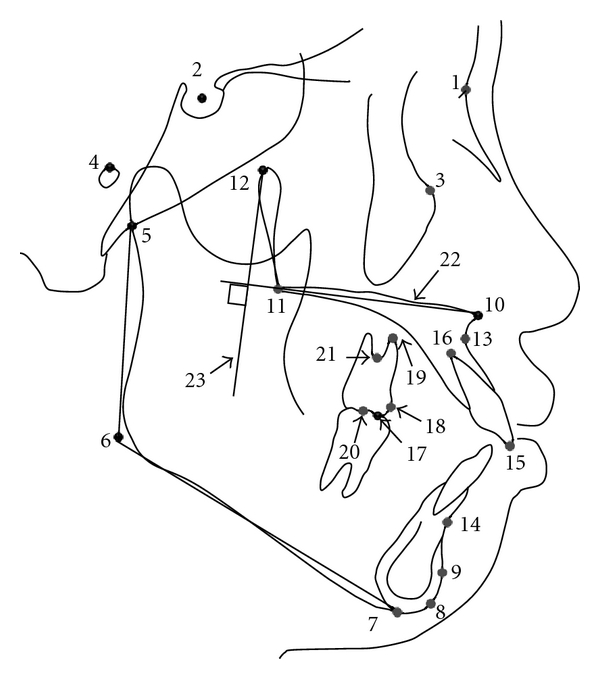
Landmarks and reference lines. (1) N: nasion; (2) S: sella; (3) Or: orbitale; (4) Po: porion; (5) Ar: articulare; (6) Go: gonion; (7) Me: menton; (8) Gn: gnathion; (9) Pog: pognion; (10) ANS: anterior nasal spine; (11) PNS: posterior nasal spine; (12) Pt: pterygoid point; (13) A: subspinale; (14) B: supramentale; (15) UIE: the incisor edge of the upper central incisor; (16) UIA: the root apex of the upper central incisor; (17) U6MB: the mesiobuccal cusp tip of the upper first molar; (18) U6M: the most mesial point of the mesial surface of the upper first molar crown; (19) U6A: the mesiobuccal root apex of the upper first molar; (20) U6C: the midpoint between the mesiobuccal and distobuccal cusps of the upper first molar; (21) U6F: the furcation point of the upper first molar; (22) Horizontal reference line, palatal plane (ANS-PNS); (23) Vertical reference line, a tangent plane to the horizontal reference plane through the Pt point.

**Figure 2:**
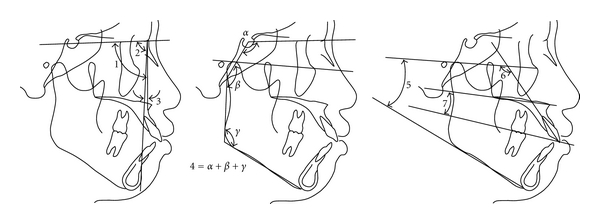
Skeletal and dental variables. (a) (1) SNA; (2) SNB; (3) ANB; (b) (4) Björk sum (*α* + *β* + *γ*); (c) (5) Frankfort mandibular plane angle (FMA); (6) U1-FH; (7) Upper occlusal plane to the palatal plane angle (UOP-PP). *α*: means the saddle angle; *β*: articular angle; *γ*: gonial angle.

**Figure 3:**
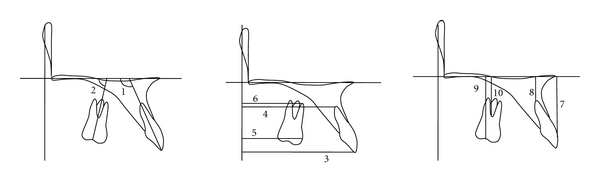
Anchorage variables. (a) (1) U1 to PP (°); (2) U6 to PP (°), (b) (3) U1E-sag: shortest distance from U1E to the vertical reference line (mm); (4) U1A-sag: shortest distance from U1A to the vertical reference line (mm); (5) U6M-sag: shortest distance from U6M to the vertical reference line (mm); (6) U6A-sag: shortest distance from U6A to the vertical reference line (mm); (c) (7) U1E-ver: shortest distance from U1E to the horizontal reference line (mm); (8) U1A-ver: shortest distance from U1A to horizontal reference line (mm); (9) U6C-ver: shortest distance from U6C to horizontal reference line (mm); (10) U6F-ver: shortest distance from U6F to horizontal reference line (mm). U1 means the long axis of the upper incisor through upper incisor edge (U1E) and root apex of the upper incisor (U1A); PP: palatal plane, horizontal reference line; U6: long axis of the upper first molar through center of the upper first molar crown on the occlusal surface (U6C) and furcation of the upper first molar (U6F); PTV: vertical reference line through the Pt point (tangent to the horizontal reference line, palatal plane); U6M: most mesial point of the mesial surface of the upper first molar crown; U6A: mesiobuccal root apex of the upper first molar.

Lateral cephalometric radiograms taken before (T0) and after treatment (T1) were traced and digitized using a graphic tablet (Wacom Co Ltd, Vancouver, BC, Canada) and V-Ceph program (Cybermed, Seoul, Korea) by one operator (YH Kim).  Figure 1 presents the landmarks and reference lines. Seven skeletal and dental (Figure 2) and ten anchorage variables (Figure 3) were measured to the nearest 0.01 mm and 0.01°.

Six randomly selected sets of cephalograms from Groups 1 and 2 were retraced and redigitized after two weeks to determine the error of measurement. There was no significant difference between the two measurements (*P* > .05; error of the linear measurement, <0.98 mm; error of the angular measurement, <1.01°). Therefore, the first measurement was used for this study. 

Mann-Whitney test was performed to compare the differences at T0 and T1 stages between two groups and to assess the amount of changes in the anchorage variables during the T0 and T1 stages between the two groups.

## 3. Results

The subjects' age, amount of crowding of the upper arch, inclination of the upper incisor to FH plane at T0 stage, and treatment duration were not different between the two groups (Table 1). The anteroposterior skeletal variables, such as SNA, SNB, ANB, APDI, and Downs' facial plane angle at the T0 stage, did not show significant difference between two groups (Table 1). However, Group 2 had a more hyperdivergent pattern than Group 1 (FMA, *P* < .05 and Björk sum, *P* < .01, Table 1). 

At the T0 stage, the anchorage variables, which describe the perpendicular distance from the upper central incisor or upper first molar to the vertical reference line (U1E-sag, U1A-sag, U6M-sag, and U6A-sag), and the angulation of the upper first molar to the horizontal reference line (U6 to PP) were not different between two groups (Table 2). 

The amounts of retraction of the upper incisor edge was significantly larger in Group 2 than Group 1 (9.5 mm : 7.1 mm, *P* < .05, Table 3). However, the upper incisor moved in a relatively controlled tipping manner in both two groups because the sagittal changes in the root apex of the upper incisor revealed 1.1 to 1.6 mm of lingual movement (Table 3). 

In terms of the posterior anchorage loss, Group 2 showed significantly less posterior anchorage loss than Group 1 (U6M-sag, 0.2 mm : 2.2 mm, *P* < .05; U6A-sag, 0.3 mm : 2.4 mm, *P* < .01; Table 3). In the vertical aspect, there was opposite vertical movement in U1 and U6 between Groups 1 and 2 : intrusion of the upper central incisor and first molar in Group 2 and extrusion of these teeth in Group 1 (U1E-ver, 0.9 mm intrusion : 0.7 mm extrusion, *P* < .05; U6F-ver, 1.0 mm intrusion : 0.9 mm extrusion, *P* < .05; Table 3).

## 4. Discussion

This study examined how much OMI could provide better posterior anchorage preservation than CAR and compared the amount of retraction of the upper anterior teeth during *en masse* retraction of the upper anterior teeth in cases with Class I malocclusion which needed maximum anchorage. 

In contrast to the subjects of the other comparative studies about the effect of OMI and CAR during *en masse* retraction of the anterior teeth [13–16], the subjects of this study were homogenous (samples with Class I malocclusion with bimaxillary dentoalveolar protrusion and minimal crowding only). Owing to the different treatment strategies and biomechanics for distalization of the upper molars in Class II malocclusion, the effect of anchorage preservation of the posterior teeth cannot be evaluated precisely in the combined samples with Class II and Class I malocclusion.

Lack of a significant difference in the treatment duration between Groups 1 and 2 (28.0 months versus 25.0 months, Table 1) suggests that OMI might not reduce the treatment duration significantly in patients who require the maximum posterior anchorage, which is in accordance with Ma et al. [16].

The more hyperdivergent pattern in Group 2 than Group 1 (Table 2) means that the OMI produced more stable posterior anchorage in this type. Patients with a hyperdivergent facial type generally have weaker natural posterior anchorage than those with a hypodivergent one [20, 21]. However, in the present study, there was significantly less posterior anchorage loss in Group 2 than Group 1 (U6M-sag, 0.2 mm versus 2.2 mm, *P* < .05; U6A-sag, 0.3 mm versus 2.4 mm, *P* < .01; Table 3). This suggests that OMI can produce superior anchorage preservation in spite of the hyperdivergent pattern.

When considering the upper premolar extraction space as 8.3 to 8.4 mm, the levels of posterior anchorage loss in Group 2 (0.2 mm, Table 3) were extremely low, whereas the levels were more than 25% of the extraction space in Group 1 (2.2 mm, Table 3). This indicates that the OMI can provide better maximum posterior anchorage than CAR. Yao et al. [13] and Lai et al. [14] also demonstrated the effect of OMI for maximum posterior anchorage loss in the treatment of maxillary dentoalveolar protrusion, and Upadhyay et al. [15] reported no anchorage loss in either the horizontal or vertical direction in mini-implants compared to conventional methods. 

In the present study, after treatment, the upper incisors were slightly more upright in the OMI group than the CAR group (U1-FH, *P* < .01, Table 2 and U1-PP, *P* < .01, Table 3), which is in accordance with Ma et al. [16].

The findings that the upper incisor showed significantly larger amounts of retraction in Group 2 than Group 1 (9.5 mm : 7.1 mm, *P* < .05, Table 3) and that the upper incisor was intruded in Group 2 and extruded in Group 1 (0.9 mm intrusion : 0.7 mm extrusion, U1E-ver, *P* < .05; Table 3) suggest that the OMI can demonstrate its ability to intrude the upper anterior teeth during retraction of the upper anterior teeth due to distal and intrusive force vector, which is in accordance with previous reports [15, 16]. 

Group 2 showed simultaneous intrusion of the upper first molar and upper central incisor (U6F-ver, 1.0 mm intrusion, *P* < .05; U1E-ver, 0.9 mm intrusion, *P* < .05, Table 3). OMI might apply a distal and intrusive vector to the entire upper arch during *en masse* retraction of the upper anterior teeth. This appears to be due to the direction of pull by the Ni-Ti closed coil spring from the OMI head to the hooks on the upper archwire and eventually can help correct the gummy smile and counterclockwise rotation of the mandible, particularly in patients with a hyperdivergent face [14]. 

Further studies will be needed to determine the effect of OMI on the anchorage preservation in various clinical situations, such as deep overbite, open bite, and severe overjet, negative overjet.

## 5. Conclusions

Although the OMI could not reduce the treatment duration, it provided better maximum posterior anchorage and greater retraction of the upper anterior teeth than CAR in spite of hyperdivergent pattern. Therefore, the null hypothesis was rejected. In addition, OMI led to an intrusion of the upper central incisor and first molar, whereas CAR resulted in extrusion of these teeth.

## Figures and Tables

**Table 1:** Demographic data of Groups 1 and 2.

			Group 1 (*N* = 20)		Group 2 (*N* = 20)		Sig.
			Mean	SD	Mean	SD	
Age at T0 stage (year)			22.16	3.11	24.64	7.85	0.1970

Dental variables at T0 stage	Crowding of the upper arch (mm)		2.15	0.80	1.83	1.07	0.2819
	UOC-PP (°)		7.63	2.11	6.91	4.70	0.5386
	U1-FH (°)		124.52	5.53	121.65	4.65	0.0827

Skeletal variables at T0 stage	Antero-posterior	SNA (°)	83.52	2.31	82.71	3.50	0.3884
		SNB (°)	81.10	1.83	79.91	3.48	0.1827
		ANB (°)	2.42	1.11	2.80	0.70	0.2070
	Vertical	FMA (°)	26.08	3.55	29.75	6.78	0.0387*
		Bjork sum (°)	393.48	3.41	398.96	7.12	0.0036**

Total treatment duration (month)		28.00	8.37	24.95	4.55	0.1602

Mann-Whitney test was performed. Group 1 means Class I malocclusion treated with conventional anchorage reinforcement; Group 2: Class I malocclusion treated with an orthodontic mini-implant; UOC: upper occlusal plane; U1: upper central incisor; SD: standard deviation; Sig.: significance; *P < .05; **P < .01.

**Table 2:** Comparison of the skeletal and dental variables before (T0) and after orthodontic treatment (T1) and the amount of change (T1-T0) between Groups 1 and 2.

			T0					T1					T1-T0				
			Group 1		Group 2		Sig.	Group 1		Group 2		Sig.	Group 1		Group 2		Sig.
			Mean	SD	Mean	SD		Mean	SD	Mean	SD		Mean	SD	Mean	SD	
Skeletal variables	Antero-posterior	SNA (°)	83.52	2.31	82.71	3.50	0.3884	83.74	3.57	81.76	3.43	0.0814	−0.21	2.31	0.95	1.04	0.0471*
		SNB (°)	81.10	1.83	79.91	3.48	0.1827	80.74	3.27	78.92	3.30	0.0875	0.36	2.49	0.99	1.13	0.3116
		ANB (°)	2.42	1.11	2.80	0.70	0.2070	3.00	1.31	2.84	1.14	0.6804	−0.58	0.73	−0.04	1.09	0.0747
	Vertical	FMA (°)	26.08	3.55	29.75	6.78	0.0387*	26.57	3.78	28.78	6.26	0.1846	−0.48	0.96	0.97	1.93	0.0046**
		Bjork sum (°)	393.48	3.41	398.96	7.12	0.0036**	394.00	3.76	397.69	6.72	0.0390*	−0.53	0.97	1.27	2.22	0.0020**

Dental variables	UOC-PP		7.63	2.11	6.91	4.70	0.5386	11.25	2.20	9.40	6.11	0.2115	−3.62	2.12	−2.49	3.99	0.2702
	U1-FH		124.52	5.53	121.65	4.65	0.0827	107.52	4.65	102.20	5.44	0.0020**	17.00	4.72	19.45	7.17	0.2106

Mann-Whitney test was performed. UOC means upper occlusal plane; U1: upper central incisor; SD: standard deviation; Sig.: significance; *P < .05; **P < .01.

**Table 3:** Comparison of the anchorage variables before (T0) and after orthodontic treatment (T1) and amount of change (T1-T0) between Groups 1 and 2.

			T0					T1					T1-T0				
			Group 1		Group 2			Group 1		Group 2			Group 1		Group 2		
	Anchorage variables		(*N* = 20)		(*N* = 20)		Sig.	(*N* = 20)		(*N* = 20)		Sig.	(*N* = 20)		(*N* = 20)		Sig.
			Mean	SD	Mean	SD		Mean	SD	Mean	SD		Mean	SD	Mean	SD	
	Angular	U1-PP	125.67	4.45	123.55	3.26	0.0934	109.47	5.93	104.43	5.77	0.0096**	16.20	5.59	19.13	6.91	0.1493
		U6-PP	86.67	4.70	83.96	4.75	0.0767	86.92	5.07	83.47	4.98	0.0360*	−0.25	3.29	0.49	4.34	0.5494

Linear	Anteroposterior	UIE	62.98	3.27	63.78	3.77	0.4784	55.89	4.18	54.33	4.48	0.2634	7.10	2.42	9.45	3.59	0.0199*
		UIA	48.77	3.06	50.37	3.23	0.1169	47.70	3.91	48.76	2.77	0.3324	1.07	1.98	1.61	1.82	0.3717
		U6M	31.45	3.53	31.87	3.29	0.6993	33.64	3.58	32.10	3.34	0.1692	−2.19	1.40	−0.24	1.62	0.0002***
		U6A	29.05	3.07	30.82	3.10	0.0779	31.46	3.09	31.08	3.15	0.6991	−2.42	1.68	−0.27	1.15	0.0000***
	Vertical	UIE	31.38	2.26	32.87	2.71	0.0666	32.02	2.25	31.98	3.04	0.9555	−0.65	1.23	0.89	1.57	0.0014**
		UIA	11.66	1.91	11.07	2.95	0.4577	10.33	2.17	8.20	2.52	0.0066**	1.33	0.96	2.88	2.03	0.0039**
		U6C	26.41	1.99	26.70	1.63	0.6227	26.58	1.88	25.76	1.95	0.1804	−0.17	1.32	0.95	1.53	0.0187*
		U6F	14.10	2.06	15.66	1.95	0.0189*	14.97	2.05	14.64	2.31	0.6288	−0.87	0.95	1.02	1.27	0.0000***

Mann-Whitney test was performed. U1 means a long axis of the upper central incisor from the incisor tip (UIE) to apex (U1A) of the upper central incisor; U6: a long axis of the upper first molar from the center of the upper first molar crown on the occlusal surface (U6C) to the furcation of the upper first molar (U6F); PP: palatal plane; UIE: incisor edge of the upper central incisor; UIA: root apex of the upper central incisor; U6M: most mesial point of the mesial surface of the upper first molar crown; U6A: mesiobuccal root apex of the upper first molar; U6C, midpoint between the mesiobuccal and distobuccal cusps of the upper first molar; U6F: furcation point of the upper first molar. Sig.: significance; *P < .05; **P < .01; ***P < .001.
